# CSB Regulates Pathway Choice in Response to DNA Replication Stress Induced by Camptothecin

**DOI:** 10.3390/ijms241512419

**Published:** 2023-08-04

**Authors:** Nicole L. Batenburg, John R. Walker, Xu-Dong Zhu

**Affiliations:** Department of Biology, McMaster University, Hamilton, ON L8S 4K1, Canada; batenbn@mcmaster.ca (N.L.B.); jwalker@mcmaster.ca (J.R.W.)

**Keywords:** Cockayne syndrome group B (CSB), camptothecin, replication stress, pathway choice, break-induced replication, alternative end joining, nonhomologous end joining

## Abstract

Topoisomerase inhibitor camptothecin (CPT) induces fork stalling and is highly toxic to proliferating cells. However, how cells respond to CPT-induced fork stalling has not been fully characterized. Here, we report that Cockayne syndrome group B (CSB) protein inhibits PRIMPOL-dependent fork repriming in response to a low dose of CPT. At a high concentration of CPT, CSB is required to promote the restart of DNA replication through MUS81–RAD52–POLD3-dependent break-induced replication (BIR). In the absence of CSB, resumption of DNA synthesis at a high concentration of CPT can occur through POLQ–LIG3-, LIG4-, or PRIMPOL-dependent pathways, which are inhibited, respectively, by RAD51, BRCA1, and BRCA2 proteins. POLQ and LIG3 are core components of alternative end joining (Alt-EJ), whereas LIG4 is a core component of nonhomologous end joining (NHEJ). These results suggest that CSB regulates fork restart pathway choice following high-dosage CPT-induced fork stalling, promoting BIR but inhibiting Alt-EJ, NHEJ, and fork repriming. We find that loss of CSB and BRCA2 is a toxic combination to genomic stability and cell survival at a high concentration of CPT, which is likely due to accumulation of ssDNA gaps, underscoring an important role of CSB in regulating the therapy response in cancers lacking functional BRCA2.

## 1. Introduction

Topoisomerase I (TOP1) plays an essential role in resolving topological stress generated by DNA replication and transcription in actively proliferating cells [1,2]. TOP1 cleaves one of the two DNA strands and becomes covalently bound to DNA, forming the TOP1 cleavage complex (TOP1cc). As soon as torsional stress on DNA is released as a result of DNA cleavage, TOP1 catalyzes DNA religation. TOP1 is specifically targeted by camptothecin (CPT) [3], which traps TOP1cc and induces single-strand DNA breaks (SSBs) [4]. These SSBs can be converted to DNA double-strand breaks (DSBs), one of the most lethal forms of DNA damage, upon encountering DNA replication forks, making CPT particularly toxic to actively proliferating cells [5]. Topotecan and irinotecan are two CPT derivatives that have been approved by the U.S. Food and Drug Administration (FDA) for treatment of colorectal, ovarian, and lung cancer [4]. However, the potency of these inhibitors is dampened by development of chemoresistance [6]. Thus, it is important to understand molecular pathways cancer cells use to respond to treatment with CPT.

Upon exposure to clinically relevant doses of CPT, replication forks rapidly slow down and undergo fork reversal [7,8]. Fork reversal, a commonly used mechanism in response to replication stress in mammalian cells, involves the coordinated annealing of nascent DNA strands, leading to the formation of a four-way structure, also known as a “chicken foot” structure [9,10]. Fork reversal is thought to protect the stability of stalled forks, allowing resumption of DNA synthesis without chromosome breakage [10,11]. Many proteins have been implicated in fork reversal [12,13], including the recombinase RAD51 [14], PARP1 [7,8], and chromatin remodelers such as SMARCAL1 [15], HLTF [16], ZRANB3 [17], and CSB [18]. The restart of CPT-induced fork reversal is dependent upon RECQ1 [8]. The resumption of DNA synthesis following CPT-induced fork stalling is also suggested to be dependent upon both active transcription and a form of break-induced replication (BIR) that is mediated by the MUS81–RAD52–POLD3 axis [19]. However, little is known about whether there are alternative pathways that can restart DNA replication following CPT-induced fork stalling, especially in the absence of BIR.

Cockayne syndrome group B (CSB) protein, encoded by the *ERCC6* gene, is a chromatin remodeler that belongs to the SNF2 family and participates in a variety of nuclear processes [12,20]. Aside from its role in transcription-coupled nucleotide excision repair [21], CSB regulates DSB repair pathway choice. CSB evicts histones from chromatin surrounding sites of DSBs to promote BRCA1-directed homologous recombination [22,23,24]. CSB possesses an intrinsic ATP-dependent fork reversal activity in vitro, albeit only when portions of its N-terminal regions are removed [18]. Although no EM studies have been conducted to date to demonstrate the fork reversal activity of CSB in vivo, several observations agree with the notion that CSB promotes fork reversal activity in vivo. CSB restrains fork progression in the presence of a low dose of hydroxyurea (HU) [18], similar to fork reversal proteins SMARCAL1, HLTF, and ZRANB3 [16,17]. CSB promotes MRE11-dependent fork degradation in BRCA1- or BRCA2-deficient cells following exposure to HU [18]. It has been well documented that fork reversal is a prerequisite for fork degradation in BRCA1- or BRCA2-deficient cells [25,26,27]. CSB is also found to promote the restart of HU-induced stalled forks through a RAD52-dependent BIR pathway in S phase [18]. A form of RAD52-dependent BIR is responsible for DNA synthesis at common fragile sites in early mitosis, known as MiDAS, following exposure to aphidicolin, an inhibitor of DNA polymerase, in S phase [28]. CSB uses its DNA translocase activity to promote MiDAS [29]. However, whether CSB regulates fork progression and fork restart following CPT-induced fork stalling has not been characterized. 

In this report, we have discovered that CSB exhibits a dose-dependent response to regulate DNA synthesis following CPT-induced fork stalling. At low concentrations of CPT (≤25 nM), CSB inhibits PRIMPOL-mediated fork repriming, likely by promoting fork reversal. On the other hand, CSB functions in the MUS81–RAD52–POLD3 axis to restart DNA synthesis in response to fork stalling induced by a high concentration of 100 nM CPT. In the absence of CSB, resumption of DNA synthesis upon 100 nM induced fork stalling does not require active transcription but relies on LIG3, LIG4, and PRIMPOL, which are inhibited by RAD51, BRCA1, and BRCA2, respectively. Our work suggests that CSB regulates pathway choice in response to CPT-induced fork stalling.

## 2. Results

### 2.1. Loss of CSB Leads to Unrestrained Fork Progression in Response to CPT at Low Concentrations

**Figure 1:**
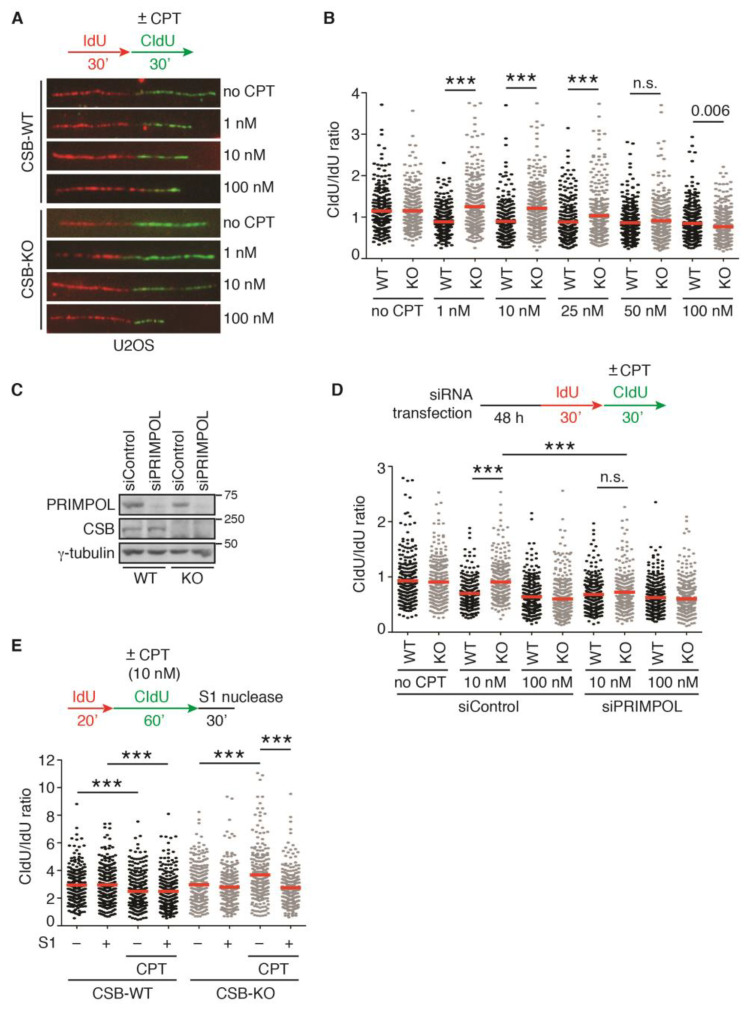
PRIMPOL mediates unrestrained fork progression in CSB-KO cells in response to a low dose of CPT: (**A**) Representative images of DNA fibers from U2OS CSB-WT and CSB-KO cells that were first labeled with IdU (red) and then labeled with CldU (green) in the presence or the absence of varying doses of CPT. (**B**) Quantification of the CldU/IdU ratio from one representative experiment. U2OS CSB-WT and CSB-KO were treated with varying doses of CPT as indicated. A total of 257–347 fibers per condition in one representative experiment were analyzed. Data from this one representative experiment are represented as scatter plot graphs, with the mean indicated in this and subsequent panels. The *p*-value was determined using a nonparametric Mann–Whitney rank-sum *t*-test in this and subsequent panels. *** *p* < 0.001. A total of two independent experiments were performed. (**C**) Western analysis of U2OS CSB-WT and CSB-KO cells transfected with siControl or siPRIMPOL as indicated. Immunoblotting was performed with antibodies against PRIMPOL, CSB, and γ-tubulin. The γ-tubulin blot was used as a loading control in this and subsequent figures. (**D**) Quantification of the CldU/IdU ratio from one experiment in which U2OS CSB-WT and CSB-KO were transfected with siControl or siPRIMPOL. A total of 234–373 fibers per condition were analyzed. *** *p* < 0.001; n.s: not significant. (**E**) Quantification of the CldU/IdU ratio in U2OS CSB-WT and CSB-KO from one experiment following treatment with S1 nuclease. A total of 255–289 fibers per condition were analyzed. *** *p* < 0.001.

It has been reported that loss of fork reversal allows resumption of DNA replication following CPT-induced fork stalling [7,8,19]. CSB has been implicated in fork reversal [18]. Therefore, we asked if CSB promotes CPT-induced fork slowing. To address this question, we performed single-molecule DNA fiber analysis in both U2OS WT and CSB-KO cells that were first labeled with IdU for 30 min and then with CldU for 30 min in the absence of CPT or in the presence of an increasing dose of CPT from 1 nM to 100 nM. Treatment with CPT, irrespective of its concentration, led to fork slowing in U2OS WT cells, as evidenced by a reduction in the ratio of CldU/IdU (Figure 1A,B), in agreement with previous finding [7]. While loss of CSB completely restored the ratio of CldU/IdU in U2OS cells treated with 1 nM or 10 nM CPT, it failed to do so in U2OS cells treated with 50 nM or 100 nM CPT (Figure 1A,B). Loss of CSB led to a partial restoration of the ratio of CldU/IdU in U2OS cells treated with 25 nM CPT (Figure 1A,B). These results suggest that CSB regulates fork progression following CPT-induced fork stalling in a dose-dependent manner. 

It has been reported that PRIMPOL-mediated fork repriming leads to unrestrained fork progression, which is associated with accumulation of ssDNA gaps [30,31,32]. To investigate whether PRIMPOL mediates fork progression in CSB-KO cells in response to CPT, we knocked down PRIMPOL in both U2OS CSB-WT and CSB-KO cells (Figure 1C). DNA fiber analysis revealed that depletion of PRIMPOL abolished restoration of the ratio of CldU/IdU in U2OS CSB-KO cells treated with 10 nM CPT, but it had no effect on the ratio of CldU/IdU in U2OS CSB-KO cells treated with 100 nM CPT (Figure 1D). The restoration of the ratio of CldU/IdU in 10 nM CPT-treated U2OS CSB-KO cells was sensitive to treatment with S1 nuclease (Figure 1E). These results suggest that unrestrained fork progression in 10 nM CPT-treated U2OS CSB-KO cells is dependent upon PRIMPOL-mediated fork repriming and is associated with ssDNA gap formation. 

### 2.2. Loss of Fork Reversal Is Insufficient to Allow Restart of DNA Synthesis in CSB-KO Cells following 100 nM CPT-Induced Fork Stalling

**Figure 2:**
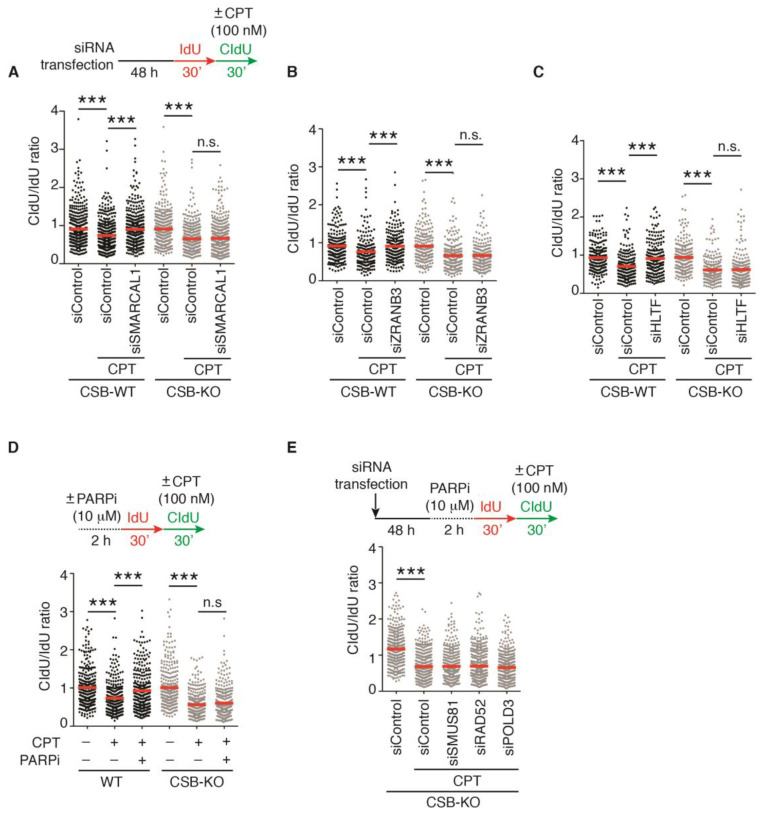
CSB is epistatic to MUS81–RAD52–POLD3 in promoting restart of DNA synthesis following 100 nM CPT-induced fork stalling: (**A**) Quantification of the CldU/IdU ratio from one representative experiment in which U2OS CSB-WT and CSB-KO were transfected with siControl or siSMARCAL1. A total of 406–428 fibers per condition in one representative experiment were analyzed. Data from this one representative experiment are represented as scatter plot graphs with the mean indicated in this and subsequent panels. The *p*-value was determined using a nonparametric Mann–Whitney rank-sum *t*-test in this and subsequent panels. *** *p* < 0.001; n.s: not significant. A total of four independent experiments were performed. (**B**) Quantification of the CldU/IdU ratio from one experiment in which U2OS CSB-WT and CSB-KO were transfected with siControl or siZRANB3. A total of 204–212 fibers per condition were analyzed. *** *p* < 0.001; n.s: not significant. (**C**) Quantification of the CldU/IdU ratio from one experiment in which U2OS CSB-WT and CSB-KO were transfected with siControl or siHLTF. A total of 206–216 fibers per condition were analyzed. *** *p* < 0.001; n.s: not significant. (**D**) Quantification of the CldU/IdU ratio from one experiment in which U2OS CSB-WT and CSB-KO were treated with PARP inhibitor olaparib and CPT as indicated. A total of 307–315 fibers per condition were analyzed. *** *p* < 0.001; n.s: not significant. (**E**) Quantification of the CldU/IdU ratio from one experiment in which U2OS CSB-KO were transfected with siControl, siMUS81, siRAD52, or siPOLD3. These cells were treated with olaparib prior to their labeling with IdU and CldU. A total of 404–450 fibers per condition were analyzed. *** *p* < 0.001.

Previously, it has been reported that loss of fork reversal enables restart of DNA replication following fork stalling induced by 100 nM CPT [19]. We have previously reported that CSB possesses an intrinsic fork reversal activity [18]. However, we have shown that CSB-KO cells, which presumably are impaired in fork reversal, fail to restart DNA synthesis following 100 nM CPT-induced fork stalling. SMARCAL1, HLTF, and ZRANB3 are chromatin remodelers that catalyze fork reversal [15,16,17]. Therefore, we investigated the possibility that fork reversal catalyzed by SMARCAL1, HLTF, and ZRANB3 might block the resumption of DNA replication in CSB-KO cells following treatment with 100 nM CPT. We knocked down these remodelers individually in both U2OS CSB-WT and CSB-KO cells. The depletion of SMARCAL1, HLTF, or ZRANB3 led to restoration of the ratio of CldU/IdU in U2OS CSB-WT cells treated with 100 nM CPT (Figure 2A–C), in agreement with a previous report that fork reversal restrains fork progression in response to this dosage [19]. However, this restoration was not detected in CSB-KO cells that were depleted for these remodelers and treated with 100 nM CPT (Figure 2A–C). To further substantiate this finding, we asked if PARP1, which is reported to maintain CPT-induced fork reversal [7], blocks restart of DNA synthesis in CSB-KO cells following 100 nM CPT-induced fork stalling. We treated both U2OS CSB-WT or CSB-KO cells with the PARP inhibitor olaparib. DNA fiber analysis revealed that while treatment with olaparib led to restoration of the ratio of CldU/IdU in U2OS CSB-WT cells treated with 100 nM CPT, in agreement with previous findings [7,8,19], it failed to do so in U2OS CSB-KO cells treated with 100 nM CPT (Figure 2D). Altogether, these results suggest that loss of fork reversal is insufficient to restart DNA replication in the absence of CSB in response to treatment with 100 nM CPT.

### 2.3. CSB Is Epistatic to MUS81/RAD52/POLD3 to Restart DNA Replication in Response to a High Dose of CPT

It has been reported that PARP inhibitor olaparib-enabled restart of DNA synthesis following CPT-induced fork stalling is mediated by a form of break-induced replication (BIR) that is dependent upon MUS81, RAD52, and POLD3 [19]. We have previously reported that CSB promotes MUS81/RAD52/POLD3-mediated BIR to restart stalled forks induced by hydroxyurea (HU) or aphidicolin [18,29]. Therefore, we asked if CSB is epistatic to MUS81, RAD52, and POLD3 in regulating fork restart following 100 nM CPT-induced fork stalling. To address this question, we first knocked down MUS81, RAD52, and POLD3 individually in U2OS CSB-KO cells. Subsequently, these cells were treated with olaparib for two hours prior to their labeling with IdU for 30 min and then with CldU for 30 min in the presence or absence of 100 nM CPT. DNA fiber analysis revealed that depletion of either MUS81, RAD52, or POLD3 had no effect on the ratio of CldU/IdU in U2OS CSB-KO cells (Figure 2E), suggesting that CSB functions through the MUS81–RAD52–POLD3 axis to promote the restart of DNA synthesis in response to a high dose of CPT.

### 2.4. Depletion of RAD51, BRCA1, or BRCA2 Restarts DNA Replication upon Treatment with 100 nM CPT in the Absence of CSB

**Figure 3:**
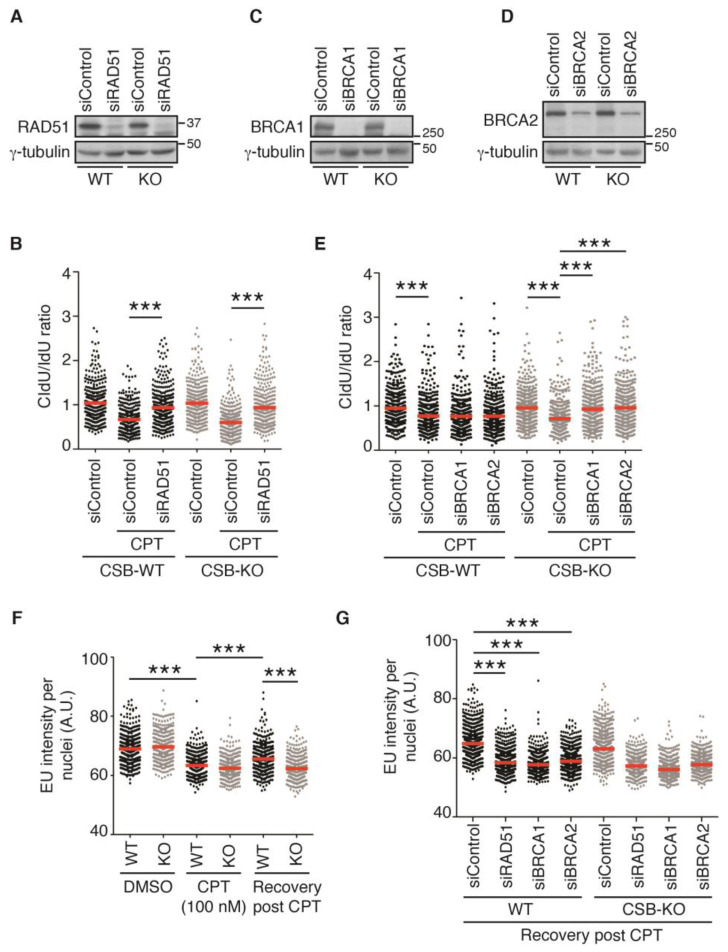
RAD51, BRCA1, and BRCA2 prevent restart of DNA synthesis following 100 nM CPT-induced fork stalling in the absence of CSB: (**A**) Western analysis of U2OS CSB-WT and CSB-KO cells transfected with siControl or siRAD51 as indicated. Immunoblotting was performed with anti-RAD51 and anti-γ-tubulin antibodies. (**B**) Quantification of the CldU/IdU ratio from one representative experiment in which U2OS CSB-WT and CSB-KO were transfected with siControl or siRAD51. A total of 359–370 fibers per condition in one representative experiment were analyzed. Data from this one representative experiment are represented as scatter plot graphs with the mean indicated in this and subsequent panels. The *p*-value was determined using a nonparametric Mann–Whitney rank-sum *t*-test in this and subsequent panels. *** *p* < 0.001. A total of two independent experiments were performed. (**C**) Western analysis of U2OS CSB-WT and CSB-KO cells transfected with siControl or siBRCA1 as indicated. Immunoblotting was performed with anti-BRCA1 and anti-γ-tubulin antibodies. (**D**) Western analysis of U2OS CSB-WT and CSB-KO cells transfected with siControl or siBRCA2 as indicated. Immunoblotting was performed with anti-BRCA2 and anti-γ-tubulin antibodies. (**E**) Quantification of the CldU/IdU ratio from one representative experiment in which U2OS CSB-WT and CSB-KO were transfected with siControl, siBRCA1 or siBRCA2. A total of 416–474 fibers per condition in this one representative experiment were analyzed. *** *p* < 0.001. A total of two independent experiments were performed. (**F**) Quantification of EU intensity from U2OS CSB-WT and CSB-KO cells from one experiment. A total of 400–483 cells per condition were analyzed. *** *p* < 0.001. (**G**) Quantification of EU intensity from U2OS CSB-WT and CSB-KO cells from one experiment. A total of 751–1037 cells per condition were analyzed. *** *p* < 0.001.

It has been reported that depletion of RAD51 restores the restart of DNA replication in cells treated with 100 nM CPT [19]. This restoration has been suggested to be attributed to the function of RAD51 in fork reversal [14,19]. Therefore, we asked if RAD51 regulates restart of DNA replication in CSB-KO cells in response to treatment with 100 nM CPT. To address this question, we knocked down RAD51 in both U2OS CSB-WT and CSB-KO cells (Figure 3A). DNA fiber analysis revealed that depletion of RAD51 restored the ratio of CldU/IdU in U2OS CSB-WT cells treated with 100 nM CPT (Figure 3A), in agreement with the previous finding [19]. Interestingly, depletion of RAD51 also restored the ratio of CldU/IdU in U2OS CSB-KO cells treated with 100 nM CPT (Figure 3A). This finding was in contrast to our earlier observation that depletion of the fork remodelers SMARCAL1, HLTF, or ZRANB3 failed to restart DNA replication in U2OS CSB-KO cells treated with 100 nM CPT. These results suggest that it is unlikely that loss of fork reversal as a result of depletion of RAD51 is a cause for restarting DNA replication in U2OS CSB-KO cells treated with 100 nM CPT. 

RAD51 is a DNA recombinase that is essential for homologous recombination (HR). Efficient loading of RAD51 to ssDNA at reversed/stalled forks is dependent upon BRCA1 and BRCA2 [33]. To investigate whether it is RAD51-dependent HR that inhibits the restart of DNA replication in response to treatment with 100 nM CPT, we knocked down BRCA1 or BRCA2 in both U2OS CSB-WT and CSB-KO cells (Figure 3C,D). DNA fiber analysis revealed that unlike RAD51, whose depletion restored the ratio of CldU/IdU in U2OS CSB-WT cells treated with 100 nM CPT (Figure 3B), depletion of either BRCA1 or BRCA2 had little impact on the ratio of CldU/IdU in U2OS CSB-WT cells treated with 100 nM CPT (Figure 3E). These results suggest that the fork reversal activity of RAD51 is responsible for preventing fork progression in U2OS CSB-WT cells treated with 100 nM CPT. Interestingly, depletion of either BRCA1 or BRCA2 restored the ratio of CldU/IdU in U2OS CSB-KO cells treated with 100 nM CPT (Figure 3E), which was similar to the depletion of RAD51 (Figure 3B), suggesting that HR proteins prevent resumption of DNA synthesis in the absence of CSB in response to treatment with a high dose of CPT.

### 2.5. Transcription Recovery Is Not Required for Restart of DNA Replication upon Treatment with 100 nM CPT in CSB-KO Cells Lacking RAD51, BRCA1, or BRCA2

It has been suggested that the restart of DNA replication following CPT-induced fork stalling requires resumption of transcription [19]. To investigate if the restart of 100 nM CPT-induced stalled forks in the absence of CSB is dependent upon transcription recovery, we measured the incorporation of 5-ethynyluridine (EU), a readout for transcription, following treatment with CPT. Treatment with CPT led to a pronounced reduction in EU incorporation in both U2OS CSB-WT and CSB-KO cells (Figure 3F). While U2OS CSB-WT cells were observed to show restoration of EU incorporation after treatment with CPT; this restoration was not detected in U2OS CSB-KO cells (Figure 3F), in agreement with a previously reported role of CSB in transcription recovery after DNA damage [34]. The depletion of RAD51, BRCA1, or BRCA2 blocked restoration of EU incorporation post release from CPT in U2OS CSB-WT cells (Figure 3G). While depletion of RAD51, BRCA1, or BRCA2 did not lead to a further decline in EU incorporation post release from CPT in U2OS CSB-KO cells, it also did not restore EU incorporation post release from CPT in U2OS CSB-KO cells (Figure 3G). Altogether, these results suggest that transcription recovery is not required for restarting DNA replication in RAD51-, BRCA1-, or BRCA2-depleted CSB-KO cells in response to treatment with a high dose of CPT. 

### 2.6. PRIMPOL Mediates Restart of DNA Replication upon Treatment with 100 nM CPT in CSB-KO Cells Depleted for BRCA2 but Not RAD51 or BRCA1

**Figure 4:**
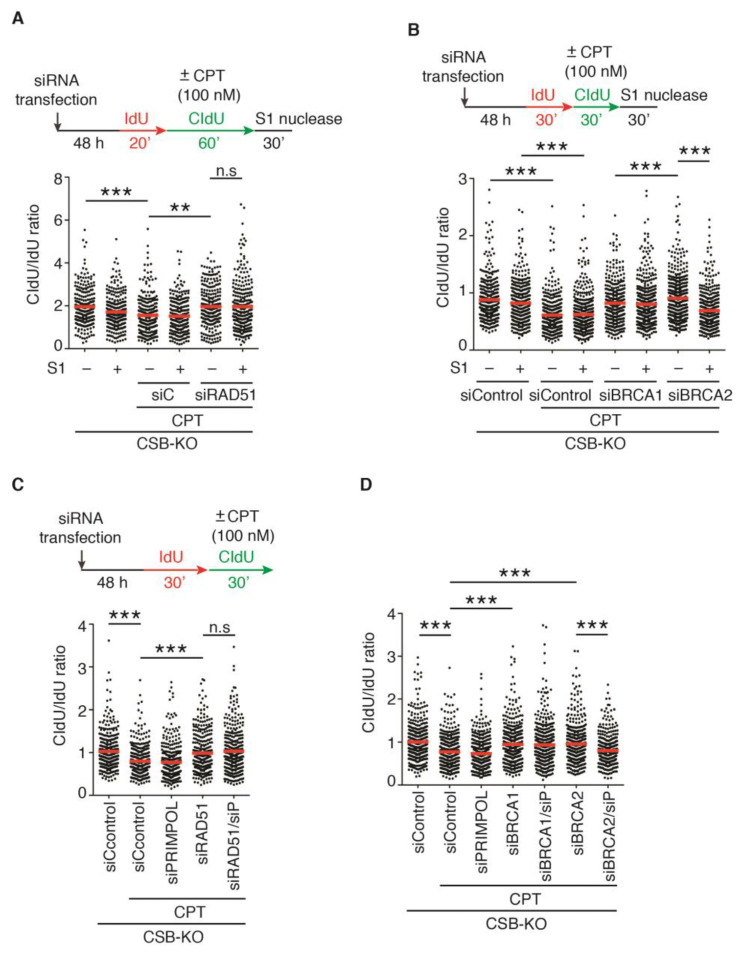
PRIMPOL mediates restart of DNA replication following 100 nM CPT-induced fork stalling in CSB-KO cells depleted for BRCA2 but not RAD51 or BRCA1: (**A**) Quantification of the CldU/IdU ratio from one experiment in which U2OS CSB-KO cells were transfected with siControl or siRAD51. DNA fiber analysis was performed following treatment with or without S1 nuclease. A total of 253–288 fibers per condition were analyzed. Data from this one experiment are represented as scatter plot graphs with the mean indicated in this and subsequent panels. The *p*-value was determined using a nonparametric Mann–Whitney rank-sum *t*-test in this and subsequent panels. ** *p* < 0.01; *** *p* < 0.001; n.s: not significant. (**B**) Quantification of the CldU/IdU ratio from one experiment in which U2OS CSB-KO cells were transfected with siControl, siBRCA1 or siBRCA2. DNA fiber analysis was performed following treatment with or without S1 nuclease. A total of 343–459 fibers per condition were analyzed. *** *p* < 0.001. (**C**) Quantification of the CldU/IdU ratio from one experiment in which U2OS CSB-KO cells were transfected with siControl, siPRIMPOL, siRAD51 or a combination of siPRIMPOL and siRAD51. A total of 324–353 fibers per condition were analyzed. *** *p* < 0.001; n.s: not significant. (**D**) Quantification of the CldU/IdU ratio from one experiment in which U2OS CSB-KO cells were transfected with siControl, siPRIMPOL, siBRCA1, siBRCA2, or siPRIMPOL in combination with either siBRCA1 or siBRCA2. A total of 391–443 fibers per condition were analyzed. *** *p* < 0.001.

RAD51, BRCA1, and BRCA2 have been implicated in ssDNA gap filling following the PRIMPOL-mediated restart of stalled replication forks [31,32,35]. Therefore, we asked if the depletion of RAD51, BRCA1 or BRCA2 leads to accumulation of ssDNA gaps in U2OS CSB-KO cells in response to treatment with 100 nM CPT. To address this question, we performed DNA fiber assays in combination with S1 nuclease following treatment with 100 nM CPT. Treatment with S1 nuclease had little effect on the ratio of CldU/IdU in either RAD51- or BRCA1-depleted U2OS CSB-KO cells upon treatment with 100 nM CPT (Figure 4A,B). In contrast, the ratio of CldU/IdU in BRCA2-depleted U2OS CSB-KO cells upon treatment with 100 nM CPT was sensitive to treatment with S1 nuclease (Figure 4B). To further substantiate this finding, we knocked down PRIMPOL in RAD51-, BRCA1- or BRCA2-depleted U2OS CSB-KO cells. DNA fiber analysis revealed that depletion of PRIMPOL abolished restoration of the ratio of CldU/IdU in BRCA2-depleted CSB-KO cells, but it had little effect on the ratio of CldU/IdU in either RAD51- or BRCA1-depleted U2OS CSB-KO cells in response to treatment with 100 nM CPT (Figure 4C,D). Taken together, these results suggest that PRIMPOL mediates the resumption of DNA replication in BRCA2-depleted but not in RAD51- or BRCA1-depleted U2OS CSB-KO cells in response to treatment with a high dose of CPT.

### 2.7. LIG3 and LIG4, Respectively, Mediate Restart of DNA Replication upon Treatment with 100 nM CPT in RAD51- and BRCA1-Depleted CSB-KO Cells

**Figure 5:**
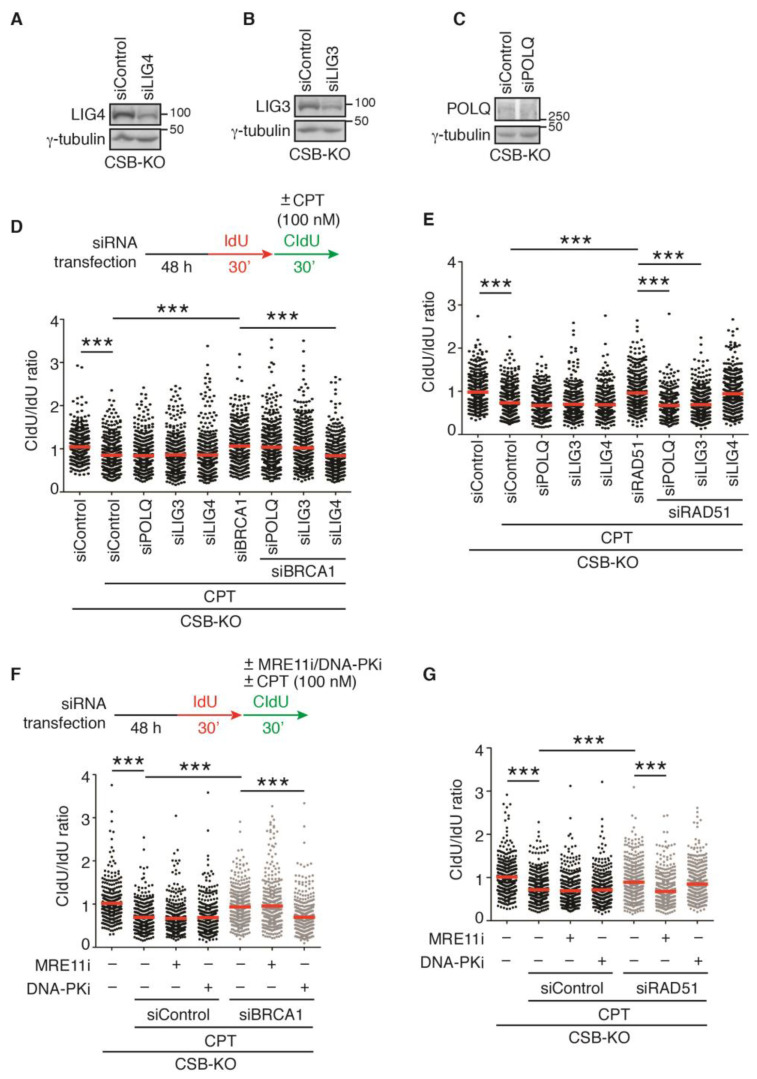
RAD51 and BRCA1 blocks respective Alt-EJ- and NHEJ-dependent restart of DNA replication in CSB-KO cells following 100 nM CPT-induced fork stalling: (**A**) Western analysis of U2OS CSB-KO cells transfected with siControl or siLIG4. Immunoblotting was carried out with anti-LIG4 and anti-γ-tubulin antibodies. (**B**) Western analysis of U2OS CSB-KO cells transfected with siControl or siLIG3. Immunoblotting was performed with anti-LIG3 and anti-γ-tubulin antibodies. (**C**) Western analysis of U2OS CSB-KO cells transfected with siControl or siPOLQ. Immunoblotting was performed with anti-POLQ and anti-γ-tubulin antibodies. (**D**) Quantification of the CldU/IdU ratio from one experiment in which U2OS CSB-KO cells were transfected with siControl, siPOLQ, siLIG3, siLIG4, siBRCA1, or siBRCA1 in combination with siPOLQ, siLIG3, or LIG4. A total of 391–451 fibers per condition were analyzed. Data from one experiment are represented as scatter plot graphs with the mean indicated in this and subsequent panels. The *p*-value was determined using a nonparametric Mann–Whitney rank-sum *t*-test in this and subsequent panels. *** *p* < 0.001. (**E**) Quantification of the CldU/IdU ratio from one experiment in which U2OS CSB-KO cells were transfected with siControl, siPOLQ, siLIG3, siLIG4, siRAD51, or siRAD51 in combination with siPOLQ, siLIG3, or LIG4. A total of 355–474 fibers per condition were analyzed. *** *p* < 0.001. (**F**) Quantification of the CldU/IdU ratio from one experiment in which siControl- or siBRCA1-depleted U2OS CSB-KO cells were treated with or without 5 μM DNA-PKcs inhibitor (DNA-PKi) or 50 μM MRE11 inhibitor (MRE11i). A total of 347–434 fibers per condition were analyzed. *** *p* < 0.001. (**G**) Quantification of the CldU/IdU ratio from one experiment in which siControl- or siRAD51-depleted U2OS CSB-KO cells were treated with or without 5 μM DNA-PKcs inhibitor (DNA-PKi) or 50 μM MRE11 inhibitor (MRE11i). A total of 437–462 fibers per condition were analyzed. *** *p* < 0.001.

CPT at a concentration of 100 nM is known to induce DSBs [7]. In the absence of HR, DSBs are repaired through error-prone pathways, such as LIG4-dependent nonhomologous end joining (NHEJ) and LIG3- and POLQ-dependent alternative end joining (Alt-EJ) [36,37]. To investigate if NHEJ and Alt-EJ pathways play a role in restarting DNA replication in CSB-KO cells in response to treatment with 100 nM CPT, we knocked down LIG3, LIG4, and POLQ individually in CSB-KO cells (Figure 5A–C). DNA fiber analysis revealed that depletion of LIG3, LIG4, or POLQ had no effect on the ratio of CldU/IdU in CSB-KO cells in response to treatment with 100 nM CPT (Figure 5D). However, depletion of LIG4 but not LIG3 or POLQ abrogated restoration of the ratio of CldU/IdU in BRCA1-depleted CSB-KO cells in response to treatment with 100 nM CPT (Figure 5D). In contrast, depletion of LIG4 did not abolish restoration of the ratio of CldU/IdU in RAD51-depleted CSB-KO cells upon treatment with 100 nM CPT (Figure 5E). Instead, restoration of the ratio of CldU/IdU in RAD51-depleted CSB-KO cells was abrogated by depletion of LIG3 or POLQ (Figure 5E). To further substantiate this finding, we measured the ratio of CldU/IdU following treatment with 100 nM CPT in the presence of either mirin, an inhibitor of MRE11 that is implicated in Alt-EJ, or NU7026, an inhibitor of DNA-PKcs that is key to NHEJ. Treatment with NU7026 but not mirin abolished restoration of the ratio of CldU/IdU in BRCA1-depleted CSB-KO cells upon treatment with 100 nM CPT (Figure 5F). On the other hand, treatment with mirin but not NU7026 abrogated restoration of the ratio of CldU/IdU in RAD51-depleted CSB-KO cells upon treatment with 100 nM CPT (Figure 5G). Taken together, these results suggest that cells lacking CSB and BRCA1 rely on LIG4-dependent NHEJ to restart DNA replication upon exposure to a high dose of CPT, whereas cells lacking CSB and RAD51 depend upon LIG3- and POLQ-mediated Alt-EJ to restart DNA replication in response to treatment with a high dose of CPT.

### 2.8. CSB and BRCA2 Are a Toxic Combination to Genomic Stability and Cell Survival in Response to Treatment with 100 nM CPT

**Figure 6:**
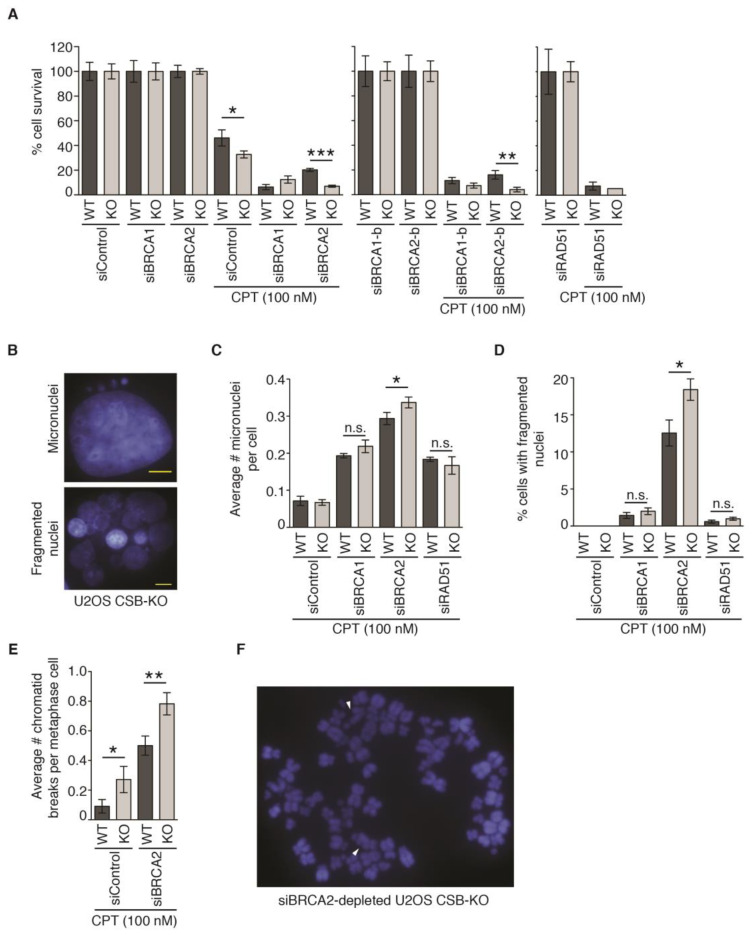
A combination of CSB and BRCA2 is toxic to genomic instability and cell survival in response to treatment with 100 nM CPT: (**A**). Clonogenic survival of U2OS CSB-WT and CSB-KO transfected with respective siRNAs as indicated. siBRCA1 and siBRCA1-b represent two independent siRNAs against BRCA1. siBRCA2 and siBRCA2-b represent two independent siRNAs against BRCA2. SDs from three independent experiments are shown in this and subsequent panels. * *p* < 0.05; ** *p* < 0.01; *** *p* < 0.001. (**B**) Representative images of micronuclei and fragmented nuclei from U2OS cells treated with 100 nM CPT. Cell nuclei were stained with DAPI in blue. Scale bar: 5 μm. (**C**) Quantification of the number of micronuclei. Forty-eight hours post transfection with indicated siRNAs, U2OS CSB-WT and CSB-KO cells were treated with 100 nM CPT for one hour and then released into fresh media for twenty four hours prior to fixation. * *p* < 0.05; n.s. not significant. (**D**) Quantification of the percentage of cells with fragmented nuclei following treatment with 100 nM CPT. U2OS CSB-WT and CSB-KO cells were transfected with indicated siRNAs. * *p* < 0.05; n.s. not significant. (**E**) Quantification of the number of chromatid breaks following treatment with 100 nM CPT. U2OS CSB-WT and CSB-KO cells were transfected with indicated siRNAs. * *p* < 0.05; ** *p* < 0.01. (**F**) Representative image of metaphase chromosome spread from siBRCA2-depleted U2OS CSB-KO cells treated with 100 nM CPT. Chromosomes were stained with DAPI in blue. White arrowheads: chromatid breaks.

To investigate if the resumption of DNA synthesis promotes cell survival in response to treatment with 100 nM CPT, we performed clonogenic survival assays in U2OS CSB-WT and CSB-KO cells depleted of RAD51, BRCA1, or BRCA2. The depletion of RAD51, BRCA1, or BRCA2 led to a severe reduction in cell survival in U2OS CSB-WT cells in response to treatment with 100 nM CPT (Figure 6A). No further change in sensitivity to 100 nM CPT was observed when RAD51 was depleted in U2OS CSB-KO cells. While a small but significant improvement in resistance to 100 nM CPT was detected in U2OS CSB-KO cells transfected with siBRCA1; this improvement was not seen with an independent siBRCA1-b (Figure 6A). In contrast, the depletion of BRCA2 by two independent siRNAs (siBRCA2 and siBRCA2-b) increased the sensitivity to 100 nM CPT in U2OS CSB-KO cells (Figure 6A). To investigate if this increased sensitivity is associated with elevated genomic instability, we measured micronuclei formation. The depletion of BRCA1, BRCA2, or RAD51 stimulated the formation of micronuclei in U2OS CSB-WT cells in response to treatment with 100 nM CPT (Figure 6B,C). Compared with U2OS CSB-WT cells, while the depletion of either BRCA1 or RAD51 did not further exacerbate the formation of micronuclei in U2OS CSB-KO cells following treatment with 100 nM CPT, the depletion of BRCA2 led to a further increase in the formation of micronuclei in U2OS CSB-KO cells following treatment with 100 nM CPT (Figure 6B,C). In addition, we observed that the depletion of BRCA2 also led to a pronounced increase in the formation of fragmented nuclei, indicative of cell death, in U2OS CSB-KO cells following treatment with 100 nM CPT (Figure 6B,D). To further substantiate this finding, we measured CPT-induced chromatid break formation in both U2OS CSB-WT and CSB-KO cells that were depleted for BRCA2. While the depletion of BRCA2 induced chromatid break formation in U2OS CSB-WT cells following exposure to CPT, this induction was further exacerbated in BRCA2-depleted U2OS CSB-KO cells following treatment with CPT (Figure 6E,F). Taken together, these results suggest that loss of both CSB and BRCA2 is a toxic combination to genomic stability and cell survival in response to treatment with 100 nM CPT.

## 3. Discussion

**Figure 7:**
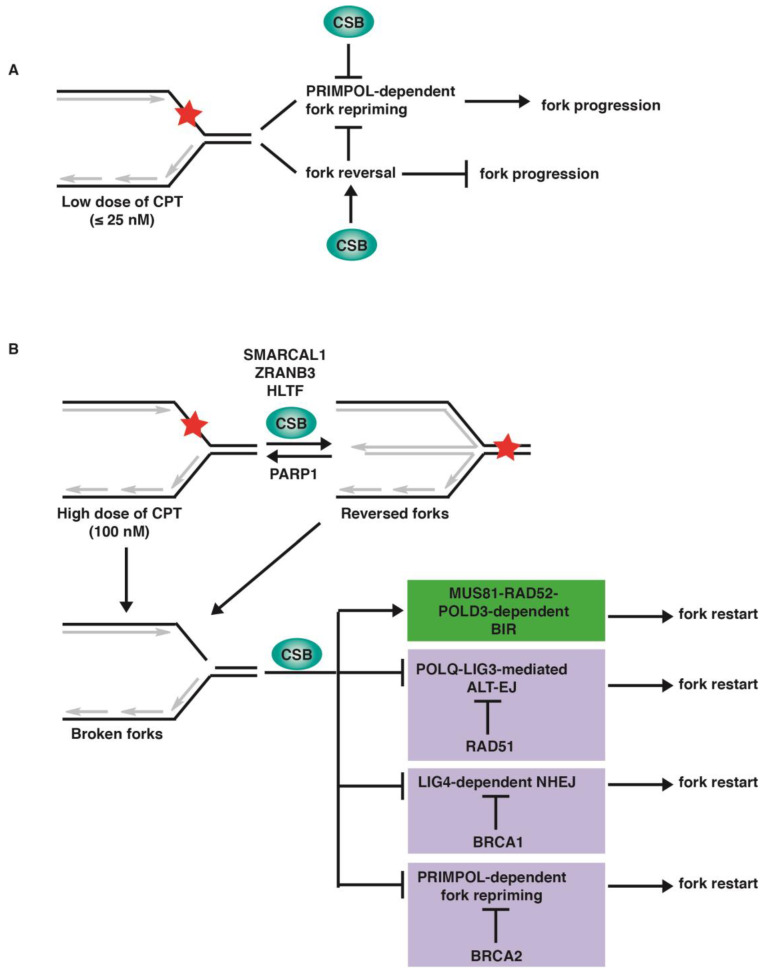
Model for the role of CSB in restraining fork progression upon a low dose of CPT, as well as controlling pathway choice to restart DNA replication in response to a high dose of CPT. See text for details. Red stars: CPT-induced DNA damage.

An understanding of how cells respond to replication stress induced by CPT is critical to optimization of chemotherapeutic strategies utilizing FDA-approved CPT derivatives. In this study, we have examined varying concentrations of CPT in the presence or absence of the fork reversal protein CSB, along with an analysis of other DNA repair proteins to demonstrate the molecular pathways involved. Our finding suggests that CSB restrains fork progression in response to a low dose of CPT, whereas it regulates pathway choice in response to a high dose of CPT, facilitating MUS81–RAD52–POLD3-dependent BIR (Figure 7).

Replication stress severity has been shown to lead to different outcomes, with increased stress leading to the generation of double-strand breaks, as well as affecting the cell cycle [38]. It has been reported that a low concentration of CPT (≤25 nM) induces reversed forks without inducing the formation of DSBs [7]. Fork reversal is thought to compete with fork repriming in response to replication stress [39,40]. PRIMPOL-mediated fork repriming leads to unrestrained fork progression that is associated with an accumulation of ssDNA gaps [31,32,35]. Our finding that PRIMPOL-dependent fork repriming is responsible for unrestrained fork progression in CSB-KO cells in the presence of ≤25 nM CPT suggests that CSB inhibits PRIMPOL-mediated fork repriming (Figure 7A). We have previously reported that CSB possesses an intrinsic fork reversal activity, albeit this activity is autoinhibited by its N-terminal region [18]. These findings suggest that CSB could inhibit PRIMPOL-dependent fork repriming through its fork reversal activity (Figure 7A), although future work is required to develop a more complete understanding of the nature of this inhibition. Our observation that loss of CSB leads to PRIMPOL-mediated unrestrained DNA replication with attendant gap formation upon exposure to a low dose of CPT raises the possibility that cancer cells deficient in CSB may be sensitive to a combination therapy of low CPT dosage and inhibitors directed against gap filling. 

At a high dosage of CPT, we showed that a deficiency in fork remodelers such as SMARCAL1, HLTF, or ZRANB3 leads to unrestrained fork progression in U2OS CSB-WT cells, yet this phenotype is abrogated in U2OS CSB-KO cells. It has been reported that loss of these fork remodelers leads to chemoresistance under certain pathological conditions [25,41]. It would be of interest to investigate whether loss of CSB may modulate chemoresistance conferred by the loss of these remodelers. 

It has been reported that 100 nM CPT induces replication–transcription collisions and R-loops [19]. The restart of CPT-induced stalled forks requires MUS81–RAD52–POLD3-mediated BIR and the subsequent transcription restart. Previously, we have reported that CSB facilitates RAD52-mediated BIR to restart HU-induced stalled forks in S phase, as well as to repair under-replicated DNA in early mitosis following exposure to aphidicolin in S phase [18,29]. Our finding that CSB is epistatic to the MUS81–RAD52–POLD3 axis suggests that CSB also facilitates BIR following CPT treatment. CSB-KO cells exhibit impaired transcription recovery following CPT treatment. Further investigation is required to parse out whether this impaired transcription recovery is due to the loss of CSB’s well-documented role in this activity or an earlier step requiring CSB in BIR.

Our finding suggests that following 100 nM CPT-induced fork stalling, CSB-KO cells, which are defective in BIR-mediated fork restart, can turn to three alternative pathways to restart DNA replication under genetic conditions lacking specific HR proteins. In these cells, restart of CPT-induced stalled forks is dependent upon POLQ-LIG3 when RAD51 is absent, whereas it is dependent upon LIG4 when BRCA1 is absent. In the absence of BRCA2, the restart of CPT-induced stalled forks in CSB-KO cells is dependent upon PRIMPOL. POLQ and LIG3 are core components of Alt-EJ, whereas LIG4 is a core component of NHEJ. These findings suggest that CSB regulates fork restart pathway choice in response to treatment with a high dose of CPT, promoting BIR but inhibiting Alt-EJ, NHEJ, and fork repriming (Figure 7B). How CSB cooperates with RAD51, BRCA1, and BRCA2, respectively, to block the three alternative pathways is unknown. We have previously reported that CSB evicts histones from chromatin surrounding sites of DSBs to regulate DSB repair pathway choice [22,23,24]. CSB has also been reported to use its DNA translocase activity to promote MiDAS, a form of RAD52-dependent BIR [29]. It is likely that CSB would also use its chromatin remodeling activity to regulate fork restart pathway choice following CPT-induced fork stalling, which requires future investigation. 

Previously, it has been reported that the depletion of BRCA2 leads to unrestrained fork progression after treatment with 1 μM CPT in U2OS cells [35]. In our study, the depletion of BRCA2 did not lead to the restart of DNA replication in the presence of 100 nM CPT in U2OS WT cells. This discrepancy is likely due to the difference in the experimental conditions since we measured DNA synthesis upon treatment with 100 nM CPT for 30 min instead of treatment with 1 μM CPT for over 6 h. Under our experimental conditions, the depletion of BRCA2 allowed the PRIMPOL-dependent restart of DNA replication in CSB-KO cells upon treatment with 100 nM CPT. This restart is associated with an accumulation of ssDNA gaps, which is likely an underlying cause of the enhanced sensitivity to 100 nM CPT in BRCA2-depleted CSB-KO cells, since replication gaps have been suggested to underlie chemosensitivity in BRCA-deficient cells [42]. The toxic combination of CSB and BRCA2 is a feature that has also been previously observed in BRCA2-depleted CSB-KO cells upon exposure to HU, olaparib, and cisplatin [18]. The work presented here lends further support to the previously claimed notion [12,43] that CSB is both a promising biomarker and a potential target in cancer treatment. 

## 4. Materials and Methods

### 4.1. Cell Culture and Transfection

All cells were grown in DMEM medium with 10% fetal bovine serum supplemented with nonessential amino acids, L-glutamine, 100 U/mL penicillin, and 0.1 mg/mL streptomycin. Cell lines used: U2OS (ATCC) and U2OS CSB-KO [22]. Cell cultures were routinely fixed, stained with DAPI, and examined for mycoplasma contamination. Transfections with siRNAs were carried out with Lipofectamine RNAiMAX (Invitrogen, MA, USA) according to its manufacturer’s instructions. 

### 4.2. Small Interfering RNAs, Antibodies, and Drugs

Small interfering RNAs (siRNAs) used were from Dharmacon: nontargeting siRNA (D-001206-14-05); siBRCA1 (D-003461-05) [18]; siBRCA1-b (CUAGAAAUCUGUUGCUAUG) [18]; siBRCA2 (D-003462-04) [18]; siBRCA2-b (J-003462-05) [18]; siHLTF (GGAAUAUAAUGUUAACGAU) [18]; siLIG3 (CAGAUAACCCAGCACAUUG) [19]; siLIG4 (GCUAGAUGGUGAACGUAGU) [19]; siMUS81 (CAGCCCUGGUGGAUCGAUA) [18]; siPOLD3 (CAACAAGGCACCAGGGAAA) [19]; siPOLQ (GGAUAAAGCUUAAUACAGA); siPRIMPOL (GAGGAAAGCUGGACAUCGA) [16]; siRAD51 (AAGGGAAUUAGUGAAGCCAAA) [19]; siRAD52 (AAGGAUGGUUCAUAUCAUGAA) [19]; siSMARCAL1 (D-013058-04-0002) [44]; and siZRANB3 (GAGUUACCUUAUUGUGAAA) [18].

Antibodies used include the following: BRCA1 (1:2000; 07-434, MilliporeSigma Canada, ON, Canada); BRCA2 (1:1000; 29450-1-AP, Proteintech, IL, USA); BrdU (1:50; 347580, BD Biosciences, ON, Canada); BrdU (BU1/75 [ICR1]) (1:400; NB500-169, Novus Biologicals, CO, USA); CSB (1:200; 10R-1587, Fitzgerald); LIG3 (1:1000; ab185815, Abcam, Cambridge, UK); LIG4 (1:500; 12695-1-AP, Proteintech); POLQ (1:200; H00010721-M09, Abnova); PRIMPOL (1:1000; 29824-1-AP, Proteintech); SMARCAL1 (1:100; sc-166209, Santa cruz, TX, USA); RAD51 (1:1000; ab63801, Abcam); and γ-tubulin (1:20,000; GTU88, Sigma, MO, USA). 

Drugs used were camptothecin (Sigma), MRE11 inhibitor mirin (Cayman Chemical, MI, USA), and DNA-PKcs inhibitor NU7026 (Sigma).

### 4.3. DNA Fiber Assay

DNA fiber analysis of replication fork progression was performed essentially as described [18]. Cells were first incubated with 25 μM IdU (I7125, Sigma) for 30 min and then 250 μM CldU (C6891, Sigma) for 30 min in the presence of 1–100 nM CPT. Following being spotted onto one end of a glass slide, cells were lysed in freshly made lysis buffer (50 mM EDTA pH 8.0, 200 mM Tris-HCl pH 7.5, 0.5% SDS) for 5 min and stretched onto the slide. Subsequently, slides were fixed in freshly made methanol:acetic acid (3:1) for 20 min at −20 °C and then allowed to air dry. Following incubation in freshly prepared 2.5 M HCl for 80 min, slides were washed three times in PBS and blocked with 5% bovine serum albumin (BSA) in PBS for 20 min at room temperature. Slides were then incubated with both rat anti-BrdU (1:800, NB500-169, Novus Biologicals) and mouse anti-BrdU (1:50, 347580, BD Sciences) antibodies prepared in 5% BSA in PBS for 1 h at 37 °C. Finally, slides were washed three times in PBS and incubated with both Alexa-488 antirat (1:250, 712-545-153, Jackson ImmunoResearch, Pennsylvania, USA) and Rhodamine antimouse (1:250, 715-295-151, Jackson ImmunoResearch) secondary antibodies for 1 h at room temperature. DNA fiber images were recorded on a Zeiss Axioplan 2 microscope with a Hamamatsu C4742-95 camera and processed in Open Lab. DNA fiber analysis was carried out with ImageJ 1.53t software (NIH).

### 4.4. S1 Nuclease Assay

To detect ssDNA replication gaps, cells were first incubated with 25 μM IdU for either 20 min or 30 min and then with 250 μM CldU for either 60 min or 30 min in the presence of CPT. Prior to DNA fiber assays, cells were pre-extracted by resuspending in 250 μL of cold CSK-100 buffer (10 mM MOPS pH 7.0, 100 mM NaCl, 3 mM MgCl_2_, 300 mM sucrose, 0.5% Triton X-100), followed by incubation on ice for 10 min and then centrifugation at 4000 rpm for 5 min. Cell pellets was resuspended in 250 μL of freshly made S1 nuclease buffer (30 mM NaAc pH 4.6, 10 mM ZnAc, 5% glycerol, 50 mM NaCl) and then incubated in the presence or the absence of 20 units/mL of S1 nuclease (EN0321, ThermoFisher) for 30 min at 37 °C. Following centrifugation at 4000 rpm for 5 min at 4 °C, cell pellets were resuspended in 200 μL of cold PBS. The preparation and imaging of DNA fibers were conducted as described above.

### 4.5. Transcriptional Recovery Assay (EU Incorporation)

To detect transcription recovery following CPT treatment, cells were either incubated for 1 h in 1 mM EU (1261-10, Click Chemistry Tools, Arizona, USA), 1 h in 1 mM EU followed by 1 h in 100 nM CPT, or 1 h in 100 nM CPT followed by washing in PBS and then treatment with 1 mM EU for 1 h. Following washing with PBS, cells were fixed in 3.7% formaldehyde in PBS for 15 min at room temperature, washed with PBS, and then incubated with 0.5% Triton X-100 in PBS for 15 min. Following washing with PBS, EU incorporation was detected using the Click-iT^®^ RNA imaging kit (C10329, Invitrogen) according to the manufacturer’s instructions. Briefly, cells were incubated with Click-iT^®^ reaction cocktail in the dark for 30 min at room temperature. Subsequently, cells were washed once with Click-iT^®^ reaction rinse buffer, once with PBS, and then stained with 4,6-diamidino-2-phenylindole (DAPI). Cell images were recorded on a Zeiss Axio Imager 2 microscope with a Zeiss Axiocam 750 monochrome camera and processed in Zeiss Zen lite. Analysis of EU incorporation was carried out with ImageJ 1.53t software (NIH).

### 4.6. Metaphase Chromosome Spreads

Metaphase chromosome spreads were prepared essentially as described [45,46]. Briefly, cells were first transfected with the indicated siRNAs. Forty-eight hours post transfection, cells were treated with 100 nM CPT for one hour, washed with PBS, and then released into fresh media for twenty hours. Subsequently, cells were arrested in 0.1 μg/mL nocodazole for 4 h and collected by trypsinization. Following wash and centrifugation, cell pellets were resuspended in 0.075 M KCl, prewarmed at 37 °C, and then incubated at 37 °C for 7 min. Cells were fixed in freshly prepared fixative (3:1 methanol:acetic acid) and kept at 4 °C for at least overnight. The fixed cells were then dropped onto glass slides and then stained with DAPI in PBS. The metaphase spreads were recorded on a Zeiss Axio Imager 2 microscope with a Zeiss Axiocam 750 monochrome camera and processed in Zeiss Zen lite.

### 4.7. Immunoblotting

Immunoblotting was performed using whole-cell extracts as described [45,47]. Briefly, cell extracts were fractionated by either 6% (for CSB, BRCA1, BRCA2, POLQ) or 8% (for PRIMPOL, RAD51, LIG3, LIG4, γ-tubulin) SDS-polyacrylamide gel electrophoresis and then transferred to nitrocellulose membranes. Following immunoblotting, membranes were exposed to Amersham hyperfilms (Cytiva 28906838, Marlborough, MA, USA). Development of the hyperfilm was performed on Konica Medical Film Processor SRX-101A, Tokyo, Japan. 

### 4.8. Clonogenic Survival Assays

Clonogenic survival assays were essentially carried out as described [18,24]. Forty-eight hours post transfection with indicated siRNAs, cells were seeded in triplicate on 6 cm plates. Four to six hours post seeding, cells were treated with 100 nM CPT for one hour. Subsequently, cells were washed with PBS and then released into fresh growth media. Ten days later, colonies were fixed and stained at room temperature for ten minutes with a solution containing 50% methanol, 7% acetic acid, and 0.1% Coomassie blue. Colonies consisting of more than 32 cells were manually scored on a microscope (Leica EZ4, Concord, ON, Canada). The number of colonies following CPT treatment was normalized to the number of colonies for untreated cells, giving rise to the percentage of cell survival. 

### 4.9. Statistical Analysis

A Student’s two-tailed unpaired t-test was used to derive all *p*-values except for where specified. 

## Data Availability

All data used in this study are available within the article or available from the authors upon request.
